# Tobacco smoking and occupational hand eczema severity in a North African adult population: a cross-sectional analytical study

**DOI:** 10.3389/falgy.2026.1723516

**Published:** 2026-05-07

**Authors:** Kacem Imène, Chouchane Asma, Ben Yahia Mounira, Aloui Aloui, Gaddour Asma, Bouhoula Maroua, Brahem Aicha, Kalboussi Houda, Chatti Souhail, Maoua Maher, El Maalel Olfa, Mrizak Nejib

**Affiliations:** 1 Faculty of Medicine of Sousse, University of Sousse, Sousse, Tunisia; 2 Occupational Medicine Department, Farhat Hached Teaching Hospital, Sousse, Tunisia; 3 Laboratoire de Recherche (LR19SP03: Etude des risques et perspectives de prévention des maladies non transmissibles en milieu professionnel), Sousse, Tunisia; 4 Occupational Medicine Department, Ibn Jazzar Teaching Hospital, Kairouan, Tunisia

**Keywords:** eczema, hand, Osnabrück hand eczema severity index score, severity, smoking

## Abstract

**Background:**

Hand Eczema (HE) is one of the most common occupational diseases worldwide. Various lifestyle factors have been implicated in HE; however, the influence of tobacco smoking on the severity of HE has not been well studied.

**Aim:**

The objective of this study is to investigate a possible association between the severity of HE and tobacco smoking.

**Methods:**

This cross-sectional analytical study included 150 patients with HE seen at the Dermatology and Allergology Unit of the Occupational Department, Farhat Hached Teaching Hospital, Sousse, Tunisia, over a period of 20 months. Data were collected using a pre-established questionnaire completed during an interview with the patient. Tobacco use was self-reported. The assessment of HE severity was based on the Osnabrück Hand Eczema Severity Index score (OHSI). Multivariate analysis was performed using multiple binary logistic regressions. Independent variables were included in the regression models when their significance level was less than 0.2.

**Results:**

In total, 150 HE patients met the inclusion criteria. The mean age of our population was 41.05 (10.3) years, with a slight female predominance (51.3% vs. 49.7%) and a sex ratio of 0.94. Almost a third of participants—or 46 (30.9%)—were smokers. The average pack years of cigarette smoking was 18.56 (12.85). The OHSI score indicated severe HE in 97 patients (64.7%). In multivariate analysis, smoking (adjusted OR: 2.83, CI: 0.99–8.14, *p* = 0.005) and perceived stress (adjusted OR: 1.06, CI: 1 −1.13, *p* = 0.01) were associated with severe HE. Similarly, having a leisure activity was inversely associated with severe HE (adjusted OR: 0.18, CI: 0.06–0.50, *p* = 0.001]).

**Conclusion:**

It is important to consider smoking cessation as an important target in the prevention and management of HE.

## Introduction

Hand eczema (HE) is a common condition in dermatology, representing 20%–35% of hand dermatoses (1). Its prevalence in the general population ranges from 2% to 8.9% of adults, and may reach up to 15%, while the incidence varies from 4.4 to 7.9 cases per 1,000 persons per year, with a median of 5.5 cases per 1,000 inhabitants per year (1, 2). HE is also one of the most frequent occupational diseases, with a reported prevalence ranging from 9% to 35% (1, 3). In Tunisia, a national study conducted in the private sector over a 10-year period reported an annual incidence of occupational allergic contact dermatitis of 31.65 cases per 100,000 workers, of which HE accounted for 87.6% of all registered cases (4).

HE includes several subtypes, namely, allergic contact dermatitis, irritant contact dermatitis, and atopic contact dermatitis (5). The consequences of this dermatosis are often underestimated. It has considerable socioeconomic and professional repercussions as well as a real impact on the quality of life of patients (6). In fact, contact dermatitis often evolves toward chronicity or recurrence (7). Severe forms affect 5%–7% of subjects and are responsible for major physical and psychological handicap (8).

HE is an inflammatory disease whose underlying mechanisms are not yet well established (1, 9). However, it is considered a multifactorial pathology, resulting from the combination of several factors such as genetic predisposition (mutation of the filaggrin gene, a structural protein of the stratum corneum), intrinsic factors (gender, age, atopy), and exogenous factors, mainly related to wet work (9). Furthermore, lifestyle factors such as eating habits, stress, alcohol consumption, and smoking have been implicated in the genesis of contact dermatitis (10). According to a Danish prospective multicenter study, the severity factors of HE include persistence into old age, its association with atopic dermatitis, and the presence of multiple contact sensitizations confirmed by patch testing (1).

Tobacco use is a major public health problem, affecting approximately 1.1 billion people worldwide, representing one-third of the world's population over the age of 15 years, according to World Health Organization (WHO) estimates (11). Tobacco has been shown to play an important role in mediating various dermatological conditions such as psoriasis, systemic lupus erythematosus, palmoplantar pustulosis, infectious eczematoid dermatitis, hidradenitis suppurativa, and allergic contact dermatitis (12). Some studies have suggested that tobacco use may promote the onset of hand dermatitis, interfere with the healing process, or directly contribute to inflammation (9).

Recently, some lifestyle factors, mainly tobacco smoking, have been reported as severity factors (9, 10, 13–15). However, existing studies dealing on the subject remain inconclusive, with contradictory results due to the diversity of the adapted methods and the studied population characteristics. To our knowledge, no Tunisian study has evaluated the association between tobacco smoking and HE severity. While the biological mechanisms linking smoking to HE severity may be universal, occupational exposures and preventive frameworks are highly context-specific. Data from North African populations remain underrepresented in international literature. Our study aimed to contribute region-specific evidence to complement global knowledge and inform tailored preventive strategies. Therefore, we conducted this study to assess the association between tobacco smoking and HE severity.

## Methods

### Study design

This was a cross-sectional analytical study that included all patients with HE seen at the Dermatology and Allergology Unit at Farhat Hached Teaching Hospital, Sousse, Tunisia, between 1 May 2018 and 31 December 2019, as part of an etiological assessment of their dermatitis.

### Participants and sampling

This study included an exhaustive sample of North African subjects aged between 18 and 65 years who were engaged in a professional activity. We included the patients who reported the notion of vesicles and itching during the progression of their eczema. Patients with doubtful histories and those who did not remember important aspects were not included in this study. Patients who did not have a lesion on their hands or in whom the diagnosis of eczema was not retained were also rejected. Finally, patients who declined participation and those with systemic diseases or users of systemic medications were also not included.

### Data collection

Data were collected using a pre-established questionnaire that was completed during patient interviews along with clinical dermatological examinations. The questionnaire consisted of three parts.

The first part explored sociodemographic data (age, gender, marital status, number of dependent children, and socioeconomic level), lifestyle habits (tobacco and alcohol consumption, type of smoking, number of pack-years, presence or not of smoking cessation, type of alcohol consumed, the number of drinks per day, and the practice of extra-professional activities), occupational characteristics (sector of activity, workstation, professional seniority, wet work, number of days of leave, and occupational repercussions) and medical data (history of atopy: atopic dermatitis, allergic rhinitis, and/or asthma, duration of the evolution of the disease, location and clinical aspect of the lesions, as well as current treatments).

The second part was related to physical examination data. During the medical interview, all the patients underwent a physical examination to record their weight and height. The degree of severity of HE was assessed by the same physician using the Osnabrück Hand Eczema Severity Index (OHSI) scoring system (16). This clinical score consisted of six morphological criteria (erythema, scaling, papules, vesicles, infiltration, and fissures), each scored on a scale from 0 to 3 according to their extent—except for fissures, graded according to their intensity. The extent of the affected area of the skin of the hands by one or more characteristic lesions was assessed by the 1/8 scoring system. For example, total palmar involvement was graded 1/8 and palmar involvement of all fingers of one hand was graded 1/8, the back of both hands 2 × 1/8 = 1/4, etc. If both hands were completely affected, the score was 8/8 = 1. Morphological aspects were then scored as follows: 0 if the lesion was absent; 1 if the extent was up to one-eighth of the area; 2 if the extent was between one-eighth and a quarter of the area; and 3 if the extent was more than a quarter of the area. Fissures were scored based on their clinical severity, which were also defined as small (<5 mm), large (≥5 mm), flat (non-bleeding), or deep (bleeding). No fissures meant a score of 0; a small flat fissure was scored as 1; numerous small flat and/or larger fissures were scored as 2; and any deep fissure was scored as 3. The final score was obtained by adding the scores of the affected areas , ranging from 0 to 18.

The third part assessed the level of the perceived stress of the participants, evaluated using the Perceived Stress Scale-10 (PSS-10) in the Arabic language (17–19), consisting of a self-administered questionnaire measuring the level of stress perceived during the past month. This version of PSS is considered to have the best psychometric qualities and remains the most economical version. The PSS-10 consisted of 10 items, six of them (item 1, 2, 3, 6, 9, and 10) considered negative and the rest (item 4, 5, 7, and 8) considered positive, reflecting the perception of the individual's capacity to face stress factors (coping). For each of the 10 items, respondents were asked how often they felt a certain way and scored on a five-point Likert scale from “never” (0) to “very often” (4). The total PSS score was the sum of all responses, ranging from 0 to 40, after inversion of the four positively stated items (19, 20).

### Operational definitions

HE was confirmed clinically by the same dermatologist for all participants based on history and physical examination. The clinical manifestations of HE include erythema, edema, vesicles, crusting, scaling, lichenification, hyperkeratosis, and fissures. The basic lesion of eczema remains vesicles (21).

Former smokers were defined as participants who had quit smoking for at least 1 year (22).

Wet work was defined as exposure to water or detergents for more than 2 h per day.

Socioeconomic status was classified as low, moderate, or high using predefined criteria aligned with WHO recommendations for health research in middle-income settings. The classification integrated three domains: educational attainment, occupational standing, and material living conditions (refrigerator, television, washing machine, car, internet), including housing tenure and access to basic utilities. Participants meeting at least two of the three domain criteria in a given category were assigned to that SES level (23).

Obesity was defined according to WHO classification as a body mass index (BMI) greater than or equal to 30 kg/m^2^ (24).

Severe HE was defined as an OHSI score of greater than 7 (25).

The perceived stress levels were classified as low if the score was less than 14, moderate for scores between 14 and 26, and high if the score was greater than or equal to 27 (19, 20).

### Data analysis

Statistical analysis was performed using IBM SPSS Statistics software (IBM Corp., Armonk, NY, USA), version 19. Normally distributed continuous variables were presented as means ± standard deviations. Categorical variables were expressed as numbers and percentages. Means were compared using Student's *t*-test. Frequencies were compared using Pearson's chi-square test. The dependent variable was HE severity. The category of non-smokers included former smokers and those who had never smoked. For multivariate analysis, multiple binary logistic regressions were performed. Independent variables were included in the regression models when their significance level was less than 0.2. Perceived stress was assessed using the PSS score and was analyzed as a continuous variable in binary logistic regression models. Odds ratios represented the change in odds of severe HE per one-unit increase in PSS score.

For all statistical tests, the significance level of the *p*-value was set at 0.05.

### Ethical consideration

Ethical approval was obtained from the institutional review board. This study adhered to the ethical principles of the Declaration of Helsinki and was conducted with respect for the rights and integrity of the participants. Informed consent was obtained from all patients for the use of their clinical data for research purposes.

## Results

**Figure 1:**
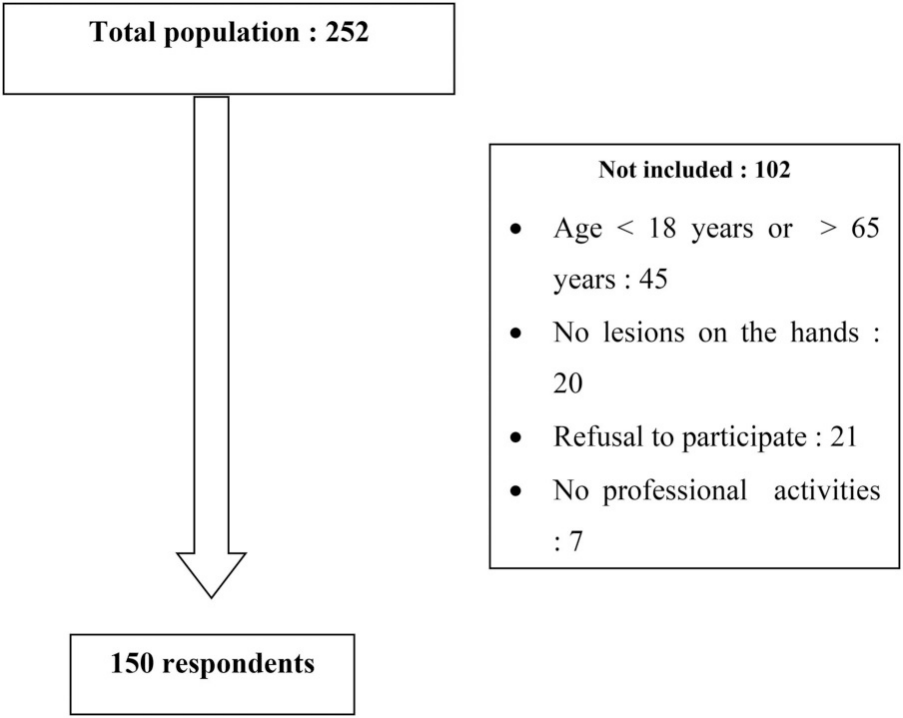
Flowchart.

In total, 150 cases of HE met the inclusion criteria (Figure 1). The mean age of our population was 41.05 ± 10.3 years, with ages ranging from 22 to 63 years. There was a slight female predominance (51.3% vs. 49.7%), with a sex ratio of 0.94.

Almost one-third of the participants (30.9% of all cases) were smokers, and 8.1% were former smokers. The mean number of pack-year (PY) smoked by the smoking participants was 18.56 ± 12.85 with a minimum of 2 PY and a maximum of 60 PY. Most smokers smoked only cigarettes (86% of the smokers), and only 12% smoked hookahs, in most cases one hookah per day. Among the employees enrolled, 113 (73.3%) had no extra-professional leisure activities. Only 15 participants (10%) practiced sports on an irregular basis.

The most represented sectors of activity were the health sector (13.3%), followed by the construction and public works sectors and the hotel industry with 12.6% each. Mean occupational seniority was 14.9 ± 10 years, with extremes ranging from 1 to 45 years. More than half of the subjects interviewed (56% of the participants) stated that they had handled irritant products in the workplace. Only 44 patients (29.3% of the participants) engaged in wet work.

In terms of medical data, the mean body mass index of the participants was 26.2 ± 4.7 kg/m^2^, with extremes ranging from 15.4 to 38.5 kg/m^2^. Obesity was observed in 20% of the participants. A personal history of allergy was found in almost one-third of the study population (41 subjects or 27.3% of cases). The “PSS-10” stress assessment found a mean score of 20.1 ± 7.8, with extremes ranging from 0 to 38 and a median of 19. Perceived stress level was considered high (score ≥ 27) in 26 participants (17.4%). HE was considered severe in 97 participants (64.7%), with a mean OHSI score of 8.44 ± 2.68 and extremes ranging from 0 to 14. Sociodemographic, occupational, medical, and lifestyle data are summarized in Table 1.

**Table 1:** Main sociodemographic, occupational, clinical, and lifestyle characteristics of the study population.

Characteristics	Number (%)
Gender	
Male	73 (48.7)
Female	77 (51.3)
Age	
18–25	13 (8.7)
26–35	37 (24.7)
36–45	50 (33.3)
46–55	36 (24)
56–65	14 (9.3)
Marital status	
Single	31 (20.7)
Married	115 (76.7)
Divorced	4 (2.7)
Number of dependent children	
≤ 3	124 (82.7)
> 3	26 (17.3)
Socioeconomic level	
Low	2 (1.3)
Medium	123 (82)
High	25 (16.7)
Education level	
Illiterate	9 (6)
<6 years	5 (33.3)
7–13 years	54 (36)
>13 years	37 (24.7)
Smoking status	
Non-smoker	92 (61.3)
Current smoker	46 (30.7)
Former smoker	12 (8)
Extra-professional activities	
Non-present	113 (75.3)
Workout	15 (10)
Gardening	7 (11.3)
Handicraft	3 (2)
Others	12 (8)
Occupational seniority (years)	
<5 years	25 (16.7)
[5–10 years]	33 (22)
>10 years	92 (61.3)
Field of activity	
Health	20 (13.3)
Building and public works	19 (12.6)
Hospitality	19 (12.6)
Clothing and textiles	17 (11.3)
Others	75 (50)
Workstation	
Worker	93 (62)
Technician	15 (10)
Executive administration	12 (8)
Others	30 (20)
Handling of irritant products	
Yes	84 (56)
No	66 (44)
Wet work	
Yes	44 (29.3)
No	106 (70.7)
Occupational repercussions	
Resumption in the same position	121 (80.7)
Redeployment	24 (15.8)
Others	5 (3.5)
Perceived stress level	
Low (PSS score < 14)	29 (19.3)
Moderate (PSS score 14–26)	95 (63.3)
High (PSS score ≥ 27)	26 (17.4)
BMI (kg/m^2^)	
<25	64 (42.7)
25–29.9	56 (337.3)
≥ 30	30 (20)
History of atopy	
Yes	41 (27.3)
No	109 (72.7)
Severity of eczema	
Non-severe (OHSI ≤ 7)	53 (35.3)
Severe (OHSI > 7)	97 (64.7)

BMI, body mass index; PSS, perceived stress scale; OHSI, Osnabruck hand eczema severity index score.

Univariate analysis revealed that male gender (OR: 1.98, CI: 1–3.9, *p* = 0.004), having three or more dependent children (OR: 2.41, CI: 1.03–5.62, *p* = 0.004), handling irritants (OR: 2.4, CI: 1.25–4.95, *p* = 0.008), perceived stress (*p* = 0.008), and smoking (OR: 2.64, CI: 1.17–5.97, *p* = 0.05) were statistically associated with the severity of HE. On the contrary, leisure time activity was associated with a significant decrease in the risk of HE severity (OR: 0, 22, CI: 0.08–0.58, *p* = 0.001). The results of the univariate analysis are provided in Table 2.

**Table 2:** Factors associated with the HE severity according to the results of the univariate analysis.

Variables	Non-severe HE (OHSI ≤ 7) (*N* = 53)		Severe HE (OHSI > 7) (*N* = 97)		*p*	OR 95% CI
	Mean (standard deviation)		Mean (standard deviation)			
Age (years)	41.3 (10.9)		40.9 (9.9)		0.8	NA
Occupational seniority (years)	15.1 (12.16)		14.8 (9.74)		0.86	NA
Pack-years number	18.4 (12.5)		19 (10.8)		0.927	NA
Perceived stress level	26.9 (6.8)		31.5 (8.6)		**0.008**	NA
	*N*	%	*N*	%		
Gender						
Female	33	62.2	44	45.4	**0**.**04**	1.98 1–3.9
Male	20	37.8	53	54.6		
Marital status						
Single	13	24.5	18	18.5	0.25	NA
Married	37	69.8	78	80.4		
Divorced	3	5.7	1	1.1		
Educational level						
Illiterate	1	1.9	8	8.2		
Elementary	16	30.2	34	35.1		NA
High school	20	37.7	34	35.1	0.25	
College	16	30.2	21	21.6		
Socioeconomic level						
Low	1	1.9	1	1		-
Medium	40	75.5	83	85.6	0.31	
High	12	22.6	13	13.4		
Number of children						
0	21	39.6	22	22.7		1
1	9	17	10	10.3	**0**.**0043**	1.060.36–3.12
2	8	15.1	27	27.8		3.221.19–8.67
3 and more	15	28.3	38	39.2		2.411.03–5.62
Physical activity						
No	45	84.9	90	92.8	0.12	NA
Yes	8	15.1	7	7.2		
Leisure activity						
No	38	71.7	89	91.7	**0**.**001**	0.22 0.08–0.58
Yes	15	28.3	8	8. 3		
Smoking status						
Non-smoker	39	73.6	53	54.6	**0**.**05**	1
Current smoker	10	18.9	36	37.1		2.641.17–5.97
Former smoker	4	7.5	8	8.3		1.470.41–5.23
Field of activity						
Health	8	15.1	12	12.4	0.10	NA
Others	45	84.9	85	87.6		
Workstation						
Worker	28	52.8	63	65	0.08	NA
Others	25	47.2	34	35		
Irritant products						
Yes	22	41.5	62	63.9	**0**.**008**	2.4 1.25- 4.95
No	31	58.5	35	36.1		
Wet work						
Yes	15	28.3	29	29.9	0.83	NA
No	38	71.7	68	70.1		
Occupational repercussions						
Resumption in the same position	14	73.7	33	86.8	0.10	NA
Redeployment	4	21.1	5	13.2		
Job modification	0	0	0	0		
Others	1	5.3	0	0		
BMI						
Normal	19	35.8	41	42.3	0.44	NA
Overweight and Obese	34	64.2	56	57.7		
History of atopy						
Yes	34	64.2	75	77.3	0.08	NA
No	19	35.8	22	22.7		

HE, hand eczema; BMI, body mass index; PSS, perceived stress scale; OHSI, Osnabruck hand eczema severity index score; CI, confident interval; NA, not applicable.

Bold values indicate statistically significant associations (*p* < 0.05).

In multivariate analysis, after controlling for confounding factors, smoking (adjusted OR: 2.83, CI: 0.99–8.14, *p* = 0.005) and perceived stress (adjusted OR: 1.06, CI: 1 −1.13, *p* = 0.01) were associated with severe HE. Similarly, having a leisure activity was inversely associated with severe HE (adjusted OR: 0.18, CI: 0.06–0.50, *p* = 0.001]). The results of the multivariate analysis are shown in Table 3.

**Table 3:** Factors associated with the HE severity according to the results of the multivariate analysis.

Variables	*p*	OR	95% CI	*p*	aOR	95% CI
Gender	0.04	1.98	1- 3.9	**ND**	**ND**	**ND**
Marital status	0.15	1.52	0.67–3.43	**ND**	**ND**	**ND**
Number of dependent children	0.004	2.41	1.03–5.62	**ND**	**ND**	**ND**
Occupational repercussions	0.10	0.53	0.12–2.27	**ND**	**ND**	**ND**
Regular physical activity	0.12	0.43	0.14–1.28	**ND**	**ND**	**ND**
Leisure activity	**0**.**001**	**0**.**22**	**0.08–0.58**	**0.01**	**0.18**	**0.06–0.68**
Smoking	**0**.**05**	**2**.**64**	**1.17–5.97**	**0.005**	**2.83**	**0.99–8.14**
Irritant products exposure	0.008	2.4	1.25–4.95	**ND**	**ND**	**ND**
History of atopy	0.08	0.5	0.25–1.09	**ND**	**ND**	**ND**
PSS score	0.008			**0.028**	**1.06**	**1–1.13**
Field of activity	0.10	0.69	0.3–2.08	**ND**	**ND**	**ND**
Workstation	0.08	1.65	0.83–3.27	**ND**	**ND**	**ND**

PSS, perceived stress scale; OR, odds ratio; CI, confident interval; ND, not determined; aOR, adjusted odds ratio.

Bold values indicate statistically significant associations (*p* < 0.05).

## Discussion

While previous research has established a relationship between smoking and HE (5, 12), the impact of smoking on HE severity remains understudied, within conclusive results. Our present study strengthens the evidence of an association between daily tobacco smoking and HE severity, aligning with prior findings (13, 14, 25). Notably, a case–control study of 230 medical laboratory technicians demonstrated a link between smoking and latex HE severity (25). Similarly, Brans et al. (13) conducted a prospective cohort study following 1,095 employees with HE for 3 years, revealing a more severe course of HE in smokers compared to non-smokers. Similarly, Olesen et al. (14) observed an association between smoking and HE severity in a cohort study of 1,491 individuals with recognized occupational HE. Their findings indicated that HE severity was higher in smokers at all six times points measured and that reducing tobacco consumption led to an improvement in patient outcome. In the literature, the link between smoking and HE has remained statistically significant regardless of whether smoking exposure was self-reported or objectively assessed (9).

However, several studies have reported contrasting results regarding the link between smoking and HE severity. A prospective study of 522 HE patients found no significant association (26), while another study of 516 housewives even reported higher HE severity in non-smokers (27).

Such discrepancies may be explained by methodological differences among studies and tobacco consumption quantification. Most published studies, including ours, rely on self-reported smoking data (13, 28), which may be unreliable. Patruno et al. (27) explored using the Fagerström test to assess tobacco dependence severity, offering a potentially more reliable approach. Notably, Lai et al. (9) employed an objective measure—serum cotinine levels—to assess tobacco exposure in active, passive, and environmentally exposed subjects. Cotinine, a nicotine metabolite, provides a more accurate and long-lasting marker of smoking compared to nicotine itself (29).

The heterogeneity of findings regarding smoking and HE severity may also be explained by neglecting clinical subtypes of HE in some studies (30). Brans and John (30) investigated this hypothesis in their study. They observed that only the vesicular eczema subtype, but not the hyperkeratotic one, was associated with self-reported smoking among patients with fairly severe occupational HE. This suggests that the relationship between smoking and HE severity may exist only for some specific subtypes of eczema.

Another explanation of these inconclusive results is the variability in eczema severity scales. In our study, the severity of HE was assessed using the Osnabrück HE Severity Index (OHSI), which includes the presence of vesicles as a severity criterion. Therefore, it is possible that the association between smoking and vesicular HE contributed significantly to the increased severity of HE in smokers compared with non-smokers. This finding is supported by Kutting et al. (31), who found that smokers with irritant hand dermatitis had significantly higher OHSI scores for erythema and vesicles than non-smokers. Moreover, chronic vesicular palmo plantar eczema is less likely to respond to PUVA therapy in smokers compared with non-smokers (32).

On the contrary, several studies have reported a dose-dependent association between smoking and the risk of developing HE (33). For example, in a study of 859 female hairdresser apprentices in Germany, Uter et al. (34) found that smoking more than five cigarettes per day was associated with HE. Similarly, another study found that smoking more than 15 cigarettes per day was associated with HE (30). Moreover, a consumption of more than 15 PY has been linked to an increased risk of contact allergy to nickel (35).

In our study, no relationship has been reported between HE severity and the number of PY consumed. Brans et al. (13) did not find an association between the number of cigarettes smoked daily and the severity of occupational HE. The authors attributed this finding to the potential unreliability of self-reported tobacco consumption data (13).

The reported associations may be explained by the inflammation induced by smoking and increased levels of pro-inflammatory agents in the body (36). Smoking can also delay wound healing, because it decreases cutaneous blood flow, causes chronic damage to the microcirculation, and inhibits fibroblast migration into lesional skin (3, 5).

Beyond tobacco smoking, perceived stress was also significantly associated with increased HE severity. These findings align with previous studies. Occupational cohorts have demonstrated associations between high perceived stress and self-reported HE (high stress associated with ∼2.5-fold higher odds) among physicians and dentists (37).

Several pathophysiological pathways may also explain this association. Chronic psychological stress activates the hypothalamic–pituitary–adrenal axis, leading to increased release of pro-inflammatory cytokines (e.g., IL-6, TNF-α) and neuropeptides that exacerbate cutaneous inflammation and impair skin barrier function (38). Stress may also promote maladaptive behaviors such as scratching, reduced treatment adherence, or increased tobacco use, which could further worsen HE (39).

In terms of leisure activities, the observed inverse association with HE severity may reflect a protective effect of stress-buffering activities on immune regulation and skin health. Engagement in leisure pursuits may improve psychological wellbeing, treatment adherence, and self-care behaviors (40). However, we acknowledge the possibility of reverse causality: Individuals with severe HE may reduce leisure participation due to pain, functional limitations, or psychosocial discomfort (41).

Our research contributes to current understanding by providing new data on the association between smoking and HE severity, while also supporting the ongoing debate. We employed validated and standardized tools to assess stress. Moreover, to provide an objective evaluation of the severity of contact dermatitis, we used the Osnabrück score, which is a clinical score grading six morphological signs according to their extension. It is simple and easy to use. The OHSI has low observer bias, as it does not rely on subjective claims. In additional, the six morphological models included in the OHSI provide a good balance between accuracy and usability (42). Finally, the OHSI scoring system has been shown to have good interobserver reliability (43).

The primary novelty of this study is that it represents, to our knowledge, one of the few investigations into the association between tobacco smoking and HE severity using the OHSI score in a Tunisian occupational population. Data from European and Asian cohorts cannot be directly extrapolated to North African settings due to distinct genetic, environmental, and occupational profiles, including specific patterns of allergen exposure. Given the high prevalence of both occupational allergen exposure and tobacco consumption among working-age adults in Tunisia, our findings highlight smoking as a critical modifiable risk factor. This emphasizes the need for tailored prevention strategies specific to our local industrial context.

However, some limitations must be considered. First, this is a case series analyses as it included only HE patients. A case–control design with a control group may allow more reliable results. Second, our study population was recruited from a single clinic that received patients referred by occupational physicians or dermatologists in the region. This may lead to a selection bias, as patients with HE may also seek care at other private, state, or semistate healthcare facilities. Furthermore, the homogeneity of the study population may limit the generalizability of our findings to other populations. The generalization of these findings to broader populations can be harder due to the limited sample size, selection according to specific criteria, and the lack of a comparison group. Thus well-designed, prospective studies with larger sample sizes are needed to confirm our observations and make generalizable conclusions. Third, data on smoking were self-reported without any objective quantification. However, bias measurement is not likely to occur because of the absence of secondary interest. Finally, despite the numerous factors analyzed in our study, additional reported factors were not considered.

## Conclusion

This study found a positive association between smoking and HE severity. Historically, addressing contact sensitization has been central to improving the prognosis of hand eczema, primarily through the cessation of occupational exposure to the offending agent. However, our study highlights that lifestyle factors, specifically tobacco smoking, can be considered a modifiable risk factor. Thus, promoting smoking cessation can improve HE prognosis. Ultimately, further research is required to elucidate the underlying mechanisms for this association.

## Data Availability

The raw data supporting the conclusions of this article will be made available by the authors, without undue reservation.
